# Pharmacological Mechanisms of Active Constituents from Traditional Chinese Medicine for Vitiligo Treatment

**DOI:** 10.3390/ph19091333

**Published:** 2026-08-24

**Authors:** Adina Hamiti, Yingying Geng, Pengfei Huang, Jinghui Xie, Tingting Cui

**Affiliations:** Xinjiang Key Laboratory of Molecular Biology for Endemic Diseases, Department of Biochemistry and Molecular Biology, Institute of Basic Medical Sciences, School of Basic Medical Sciences, Xinjiang Medical University, Urumqi 830017, China; adina@stu.xjmu.edu.cn (A.H.); yingyingg@stu.xjmu.edu.cn (Y.G.); hpf123690@163.com (P.H.)

**Keywords:** vitiligo, traditional Chinese medicine, oxidative stress, immunomodulation, neuro–immune–skin axis

## Abstract

Vitiligo is a chronic acquired depigmenting disorder characterized by progressive melanocyte loss. Its pathogenesis involves a multifactorial interplay among oxidative stress, impaired melanocyte regeneration, immune-mediated cytotoxicity, and neuroendocrine dysregulation. In recent years, traditional Chinese medicine (TCM)-derived active ingredients have attracted increasing research interest as potential interventions for vitiligo, although most isolated compounds remain at the preclinical stage. This review summarizes pharmacological and natural compounds applied in vitiligo treatment and highlights their regulatory mechanisms on core pathogenic modules. A comprehensive analysis of natural and semisynthetic active ingredients is provided, covering their preclinical and clinical evidence, mechanisms of action, and therapeutic relevance. Multiple ingredients, including baicalein, quercetin, curcumin, paeoniflorin, kaempferol, glycyrrhizin, and epigallocatechin-3-gallate (EGCG), have demonstrated antioxidant, anti-inflammatory, immunomodulatory, and neuroprotective effects that are involved in the pathogenesis of vitiligo. Some TCM-derived interventions, such as *Ginkgo biloba* L. extract and compound glycyrrhizin, have been evaluated clinically, whereas most isolated active compounds, including baicalein and quercetin, remain at the preclinical stage. Overall, TCM-derived active ingredients represent a promising therapeutic strategy for vitiligo. However, continued translational and clinical research is still required to optimize formulations, dosing regimens, and safety profiles, thereby facilitating their integration into routine clinical practice.

## 1. Introduction

Vitiligo is an acquired pigmentary skin disorder affecting 0.5–2% of the global population. It is characterized by progressive loss of melanocytes and formation of white patches on the skin, which significantly impacts patients’ quality of life, often leading to social stigma, anxiety, and reduced self-esteem [1]. The pathogenesis of vitiligo is heterogeneous and involves interactions among genetic susceptibility [1], environmental factors such as oxidative stress [2,3,4,5], chemical exposure [6], immune-mediated melanocyte destruction [7,8], impaired melanocyte adhesion and regeneration [1], and neuroendocrine factors [1]. Oxidative stress contributes to melanocyte injury in vitiligo by impairing the Nrf2-mediated antioxidant system and reducing the activity of enzymes, including catalase (CAT) and glutathione peroxidase (GSH-Px) [2,3,4,5]. Certain chemicals, particularly phenolic and catecholic compounds, may cause chemical leukoderma or trigger vitiligo-like depigmentation [6]. Damaged melanocytes expose autoantigens, activating CD8^+^ T cells, which in turn produce inflammatory cytokines and promote targeted melanocyte destruction [7,8]. Concurrently, surrounding keratinocytes secrete abundant chemokines that further enhance T-cell recruitment, while psychological stress amplifies local inflammation via the neuro–immune–skin axis [9,10]. The synergistic effects of multiple targets collectively make vitiligo a difficult-to-treat skin disease.

In recent years, bioactive compounds derived from traditional Chinese medicine (TCM) have emerged as promising candidates due to their multi-target properties. Extensive evidence has suggested their potential anti-vitiligo effects, such as curcumin [11,12], epigallocatechin-3-gallate [13,14], and other constituents. Numerous studies have also elucidated their mechanisms of action. Baicalein restores redox balance via Nrf2-mediated antioxidant signaling [15]. Quercetin inhibits melanocyte ferroptosis through the Nrf2/glutathione peroxidase 4 (GPX4) axis [16]. Demethylzeylasteral reduces immune-mediated cytotoxicity by suppressing JAK/STAT signaling [17]. 3,5-Dicaffeoylquinic acid promotes melanin synthesis in B16 cells through Wnt/β-catenin pathway activation [18]. Although existing studies have elucidated the therapeutic applications and potential mechanisms of various TCM active compounds in vitiligo, a systematic synthesis of this evidence remains lacking.

Current first-line treatments for vitiligo include topical corticosteroids, calcineurin inhibitors, NB-UVB phototherapy, and recently JAK inhibitors (e.g., ruxolitinib) [19,20,21,22]. Topical corticosteroids and calcineurin inhibitors suppress local T-cell activation and inflammatory cytokine production [1,23], while NB-UVB promotes Treg induction, melanocyte migration, and modulation of the inflammatory microenvironment [24,25]. JAK inhibitors block IFN-γ signaling and reduce CXCL10-mediated recruitment of autoreactive CD8^+^ T cells [23,26]. Notably, TCMs share certain immunomodulatory properties with these conventional agents, including regulation of T-cell subsets and suppression of pro-inflammatory cytokines [17,27,28,29,30,31]. However, TCMs offer distinctive advantages that are not typically observed in first-line therapies, such as potent antioxidant effects via Nrf2 pathway activation [15,32,33,34], direct promotion of melanogenesis through tyrosinase/MITF upregulation [18,35], and multi-target holistic regulation that addresses the heterogeneous pathogenesis of vitiligo [27,36,37,38]. These unique properties position TCMs as a valuable therapeutic strategy with the potential to achieve more sustained repigmentation and improved outcomes, particularly when integrated with existing treatment modalities.

In this review, we first summarize the clinical evidence for TCM-derived bioactive compounds in vitiligo treatment; then, we discuss their regulatory mechanisms on vitiligo pathogenic targets, including the modulation of redox balance, immune responses, melanocyte regeneration, and neuro–immune–skin interactions. Based on these findings, we construct a therapeutic landscape centered on vitiligo disease targets, illustrating the multi-target actions of bioactive compounds. This review aims to provide a strategic reference for future TCM-based drug development for vitiligo.

## 2. Methods

This review was conducted as a review. The literature was identified through systematic searches of the PubMed and Web of Science, covering publications from January 2000 to July 2026. Search terms included combinations of the following keywords: “vitiligo”, “leukoderma”, “melanocyte”, “autoimmunity”, “melanogenesis”, “pigmentation”, “repigmentation”, “traditional Chinese medicine”, “Chinese herbal medicine”, “Uyghur medicine”, “medicinal plant”, “herbal formula”, “botanical extract”, “natural product”, “bioactive compound”, “oxidative stress”, “Nrf2”, “mitochondrial dysfunction”, “autophagy”, “ferroptosis”, “immune regulation”, “JAK/STAT”, “chemokine”, and “neuro-immune-skin axis”. Only English-language publications were considered for inclusion. Eligible publications included original research articles, clinical trials, observational studies, and peer-reviewed review articles. Conference abstracts, dissertations, books, editorials, and commentaries were excluded.

Titles and abstracts were screened independently by two authors, and full texts were retrieved for potentially relevant articles. Disagreements were resolved through discussion or consultation with a third author. Additional relevant publications were identified by manually screening the reference lists of included articles. The final selection was based on relevance to the scope of this review, prioritizing high-quality evidence (e.g., randomized controlled trials for clinical aspects and mechanistic studies with robust experimental design for basic research).

We acknowledge that as a review, our selection was not exhaustive and may be subject to selection bias; therefore, we have revised our language throughout the manuscript to present our findings as an overview of representative evidence rather than a complete or comprehensive summary of the entire field. Negative or inconclusive findings have been included where available to provide a balanced perspective.

## 3. Clinical Translation and Evidence-Based Advances

### 3.1. Human Clinical Evidence

#### 3.1.1. Topical Therapy

A small randomized, double-blind, placebo-controlled pilot study reported that topical turmeric cream reduced lesion size and improved lesion appearance after four months of treatment [11], but another systematic review on curcumin did not establish curcumin as a validated photosensitizer for vitiligo-specific phototherapy [12]. These data support curcumin/turmeric as a candidate adjunct, but not yet as a clinically established sensitizer for phototherapy.

Chemical modification offers a strategy to overcome the pharmacokinetic limitations of natural polyphenols, particularly low lipophilicity and poor transdermal permeability. Peracetylation has been shown to increase epigallocatechin-3-gallate (EGCG) bioavailability, and AcEGCG is rapidly converted intracellularly to EGCG [39]. Topical 0.5% AcEGCG cream was associated with repigmentation and stabilization of depigmentation in two patients with vitiligo [14]. A clinical study comparing topical EGCG with topical pimecrolimus detected no statistically significant between-group difference in VASI improvement [13]. However, because the study was not designed as an equivalence or non-inferiority trial, the absence of a statistically significant difference does not establish equivalent or comparable efficacy.

In addition to these antioxidants, natural plant-derived oils may be useful in the treatment of anatomically sensitive areas. *Nigella sativa* L. seed oil, rich in thymoquinone, has antioxidant and immunomodulatory activity. A clinical study evaluating topical *Nigella sativa* L. seed oil cream for vitiligo reported repigmentation in facial, acral, and genital regions. Notably, in genital areas, ≥50% repigmentation was achieved in seven of eight patients, with no adverse effects reported during the 6-month study; however, the small sample size and lack of a placebo group preclude firm conclusions regarding safety [40].

#### 3.1.2. Systemic Therapy

A randomized double-blind trial and an open-label pilot study collectively support *Ginkgo biloba* L. extract (EGb) as a systemic antioxidant adjunct in vitiligo management, demonstrating its ability to halt disease progression and induce repigmentation in some patients [41,42]. Compound glycyrrhizin (CG) has accumulated substantial clinical evidence as an immunomodulator in vitiligo. A meta-analysis of 39 randomized controlled trials (RCTs) involving 3,994 patients reported improved repigmentation with CG-containing combination therapy, while pooled adverse-event incidence was not increased (RR = 0.79, 95% CI: 0.62–1.00, P = 0.05); however, long-term safety remains insufficiently established [43]. A recent RCT further supports CG as a potential steroid-sparing strategy for vitiligo in children. In this comparative study, oral CG (OCG, 50–150 mg/d) and oral prednisone (OP), each combined with phototherapy, both produced VASI improvement at week 52, and no statistically significant between-group difference was detected. However, given the small sample size (n = 50) and limited follow-up, these findings indicate short-term tolerability rather than established long-term safety in children [44]. In a retrospective study, adding TGP improved clinical outcomes, including greater repigmentation in head and neck lesions and reduced serum oxidative stress markers, compared with NB-UVB plus corticosteroid alone [45]. However, the independent contribution of TGP requires prospective validation.

Oral TCM preparations have been evaluated in both clinical and preclinical settings. In a prospective study of 233 patients with segmental vitiligo, Yiqi Qubai granules combined with a 308 nm excimer laser produced higher response and repigmentation rates than either treatment alone [46]. By contrast, a single case report described spontaneous repigmentation during regular consumption of a turmeric-and-honey beverage; however, concurrent reductions in psychological stress, adoption of a vegetarian diet, and avoidance of alcohol preclude causal attribution to curcumin [47]. Table 1 summarizes the available human clinical evidence for the topical, systemic, and combined TCM-derived interventions discussed in this section.

### 3.2. Preclinical Evidence

Apigenin is another promising candidate supported by evidence across multiple experimental systems. In a hydroquinone-induced vitiligo model, topical apigenin at concentrations of 1%, 2.5%, and 5% ameliorated depigmentation, reduced oxidative stress and inflammatory markers, and increased the number of melanin-containing hair follicles, with efficacy comparable to tacrolimus [48]. A recent study using zebrafish and human skin explant showed that apigenin reversed phenylthiourea (PTU)-induced depigmentation in zebrafish and increased melanin content in human skin tissue by approximately 1.3-fold. These data provide pharmacodynamic support for developing topical formulations with dual pro-melanogenic and photoprotective properties [35]. Artiri La Li Honey Pill (ALLHP) showed antioxidant and pro-melanogenic effects in animal and zebrafish models by increasing melanin content and tyrosinase levels and reducing malondialdehyde [49]. However, these ALLHP findings remain preclinical and require clinical validation. Representative preclinical and translational evidence, together with the corresponding experimental models and translational limitations, is summarized in Table 2.

## 4. Mechanisms of TCM Ingredients in Vitiligo

### 4.1. Remodeling Redox Homeostasis

Oxidative stress is considered an important driver in the pathogenesis of vitiligo [50]. Such stress commonly causes melanocyte damage, including mitochondrial dysfunction, impaired autophagic flux, ferroptosis, endoplasmic reticulum (ER) stress, and apoptosis. For TCM-derived ingredients, the central question is whether they can restore endogenous stress-response systems in melanocytes and in the surrounding epidermal microenvironment.

#### 4.1.1. Targeting Nrf2 to Enhance Antioxidant Defense

Nuclear factor erythroid 2-related factor 2 (Nrf2) is a central mediator of cellular resistance to oxidative stress [51]. Under physiological conditions, Kelch-like ECH-associated protein 1 (Keap1) anchors Nrf2 in the cytoplasm for proteasomal degradation. Upon oxidative stress, dissociation of the Keap1/Nrf2 complex protects cells against mitochondrial dysfunction [52]. However, melanocytes in vitiligo exhibit functional defects in the Nrf2-antioxidant response element (ARE) pathway, impairing ARE-mediated transcription [53]. Therefore, reactivating the Nrf2-ARE/heme oxygenase-1 (HO-1) axis is a key strategy for restoring melanocyte antioxidant capacity. TCM active ingredients with diverse chemical structures may feed into this pathway through different routes.

Pharmacological rescue experiments supported the involvement of these pathways. Polyphenolic compounds and flavonoids protect melanocytes from oxidative injury mainly through the Nrf2/ARE antioxidant defense axis. Paeonol, baicalein, and apigenin activate Nrf2 signaling in melanocytes, leading to increased expression of antioxidant enzymes, such as HO-1, superoxide dismutase 2 (SOD2), CAT, and GSH-Px, while reducing MDA accumulation [15,32,33]. In addition, kaempferol activates Nrf2/ARE-regulated genes, including HO-1, glutamate-cysteine ligase catalytic subunit (GCLC), and NAD(P)H quinone dehydrogenase 1 (NQO1) in HaCaT keratinocytes, suggesting that flavonoids may also support melanocyte survival by improving the epidermal oxidative microenvironment [54]. Collectively, these studies position the Nrf2/ARE axis as a well-supported antioxidant target, though the strength of evidence varies by compound and model system.

Paeoniflorin, a terpene glycoside derived from *Paeonia lactiflora* Pall., regulates Nrf2 signaling via multiple mechanisms. First, through the c-Jun N-terminal kinase (JNK)-dependent pathway, paeoniflorin increased JNK phosphorylation without significantly affecting p38 or extracellular signal-regulated kinase (ERK) in H_2_O_2_-treated PIG1 and PIG3V human melanocyte models. Inhibition of JNK by its inhibitor SP600125 suppresses paeoniflorin-induced Nrf2 nuclear translocation and HO-1 expression, supporting a role for JNK as an upstream kinase [34]. Second, recent research highlights a non-canonical mechanism involving crosstalk between the cytoskeletal adapter protein PDZ and LIM domain protein 1 (PDLIM1) and the RhoA/ROCK1 (Ras homolog family member A/Rho-associated coiled-coil containing protein kinase 1) axis. PDLIM1 deficiency exacerbates oxidative sensitivity, whereas paeoniflorin confers protection by restoring the PDLIM1-Nrf2 axis [55], linking cytoskeletal dynamics to antioxidant defense in melanocytes.

Standardized botanical extracts may provide broader cytoprotection through the combined actions of multiple constituents. *Ginkgo biloba* L. extract (EGb761) was associated with activation of the Nrf2/ARE pathway in melanocytes [56], significantly elevating cellular resistance to oxidative stress. Importantly, these protective effects extend beyond melanocytes to fibroblasts and endothelial cells, potentially reducing UVB-induced cytotoxicity and inflammatory cascades at the tissue level [57,58].

#### 4.1.2. Regulating Mitochondrial Homeostasis and Autophagy

Mitochondrial dysfunction and impaired autophagic flux represent additional core pathogenic mechanisms of melanocyte oxidative damage [59,60]. Melanocytes in vitiligo exhibit autophagic dysregulation, rendering them hypersensitive to hydrogen peroxide (H_2_O_2_)-induced oxidative stress [61]. Polyphenols and flavonoids primarily inhibit mitochondria-dependent apoptotic pathways. Epigallocatechin-3-gallate (EGCG), a green tea-derived catechin from *Camellia sinensis* (L.) Kuntze, and its semisynthetic derivatives protect melanocytes against oxidative injury. In H_2_O_2_-treated PIG1 human melanocytes, pretreatment with EGCG or the semisynthetic caffeic acid derivative WSY6 (6.25, 12.5, or 25 μM for 1 h), followed by exposure to 1 mM H_2_O_2_ for 1 h, dose-dependently reduced oxidative cytotoxicity, with WSY6 exhibiting greater antioxidant potency [62]. In H_2_O_2_-stressed human epidermal melanocytes, AcEGCG reduced ROS generation and p38 MAPK phosphorylation, restored mitochondrial membrane potential, and attenuated apoptosis [63]. WSY6 limits cytochrome c release from mitochondria, thereby preventing downstream caspase-9 and caspase-3 activation [62]. Similarly, in PIG1 human melanocytes pretreated with baicalein (10, 20, or 40 μM) for 1 h before exposure to 1.0 mM H_2_O_2_ for 24 h, baicalein concentration dependently reduced p38 MAPK phosphorylation, preserved mitochondrial membrane stability, and attenuated apoptosis, while ERK phosphorylation was not significantly affected [64].

Beyond single-compound treatment, alkaloids suggest a possible route for organelle repair. Mesenchymal stem cells (MSCs) are promising candidates for autoimmune dermatoses due to their immunomodulatory and tissue-repair capacities [65]. Preclinical studies show that MSCs modulate the phosphatase and tensin homolog (PTEN)/phosphoinositide 3-kinase/protein kinase B (PI3K/AKT) pathway to influence melanocyte proliferation and apoptosis, while also regulating cytokine secretion and T-cell balance [66]. Capsaicin, the primary pungent alkaloid in chili peppers (*Capsicum* spp.) [67], has been evaluated in combination with MSCs. A recent study showed that combined treatment with capsaicin and MSCs improved mitochondrial function in PIG3V melanocytes and was associated with inhibition of the heat shock protein 70 (HSP70)/Toll-like receptor 4 (TLR4)/mechanistic target of rapamycin (mTOR)/focal adhesion kinase (FAK) signaling axis [68]. These findings indicate an enhanced combined-treatment effect rather than confirmed pharmacological synergy.

Peng et al. investigated Lycium barbarum polysaccharide (LBP) in PIG1 human melanocytes exposed to H_2_O_2_. LBP increased the expression of autophagy-related proteins, including Beclin 1, LC3-II, and Atg5, and increased autophagic vacuoles observed by transmission electron microscopy. Rapamycin enhanced, whereas 3-MA attenuated, the LBP-associated increase in Beclin 1 expression and melanocyte viability. Nrf2 overexpression and knockdown experiments further supported the involvement of the Nrf2/p62 pathway in these autophagy-associated and cytoprotective effects [69].

#### 4.1.3. Modulating Ferroptosis-Related Processes and Endoplasmic Reticulum Stress

Beyond traditional apoptosis, ferroptosis and ER stress are increasingly recognized as key contributors to melanocyte loss in vitiligo [60]. Ferroptosis is an iron-dependent, lipid peroxidation-mediated form of regulated cell death closely linked to redox imbalance. In vitiligo, oxidative stress may promote iron dyshomeostasis, glutathione depletion, lipid ROS accumulation, and GPX4 dysfunction, thereby increasing melanocyte susceptibility to ferroptosis [16]. Moreover, interferon-gamma (IFN-γ), a central autoimmune mediator in vitiligo, can synergize with oxidative stress to aggravate glutathione depletion and lipid peroxidation, further amplifying ferroptosis-related melanocyte damage [70]. Pharmacological targeting of this immunity-metabolism crosstalk may therefore provide a potential therapeutic strategy for vitiligo.

Several TCM-derived ingredients have been reported to modulate ferroptosis-associated iron dysregulation. In an RSL3/FAC-treated human melanocyte injury model, baicalein reduced transferrin receptor 1 (TFR1) expression, limited intracellular iron accumulation and lipid ROS, and increased GPX4 expression, changes consistent with attenuation of ferroptosis-related injury [71]. In erastin-treated melanocytes, curcumin reduced iron accumulation and MDA, restored GSH and SOD levels, increased GPX4, and decreased acyl-CoA synthetase long-chain family member 4 (ACSL4) and TFR1; ferrostatin-1 (Fer-1) similarly rescued erastin-induced injury, providing functional support for ferroptotic cell death in this model. Curcumin-mediated protection was associated with Nrf2/HO-1 activation, and Nrf2 inhibition with ML385 attenuated this effect [72]. Similarly, quercetin reduced lipid peroxidation and ferroptosis-associated changes in hydroquinone-induced guinea pig and B16 cell models; partial loss of protection after Nrf2 knockdown supported the involvement of the Nrf2/GPX4 axis [16]. In RAS-selective lethal 3 (RSL3)-induced models, kaempferol reduced the labile iron pool and lipid ROS and increased GPX4, SOD, and CAT; loss of protection after GPX4 knockdown supported GPX4-dependent cytoprotection [73].

**Figure 1:**
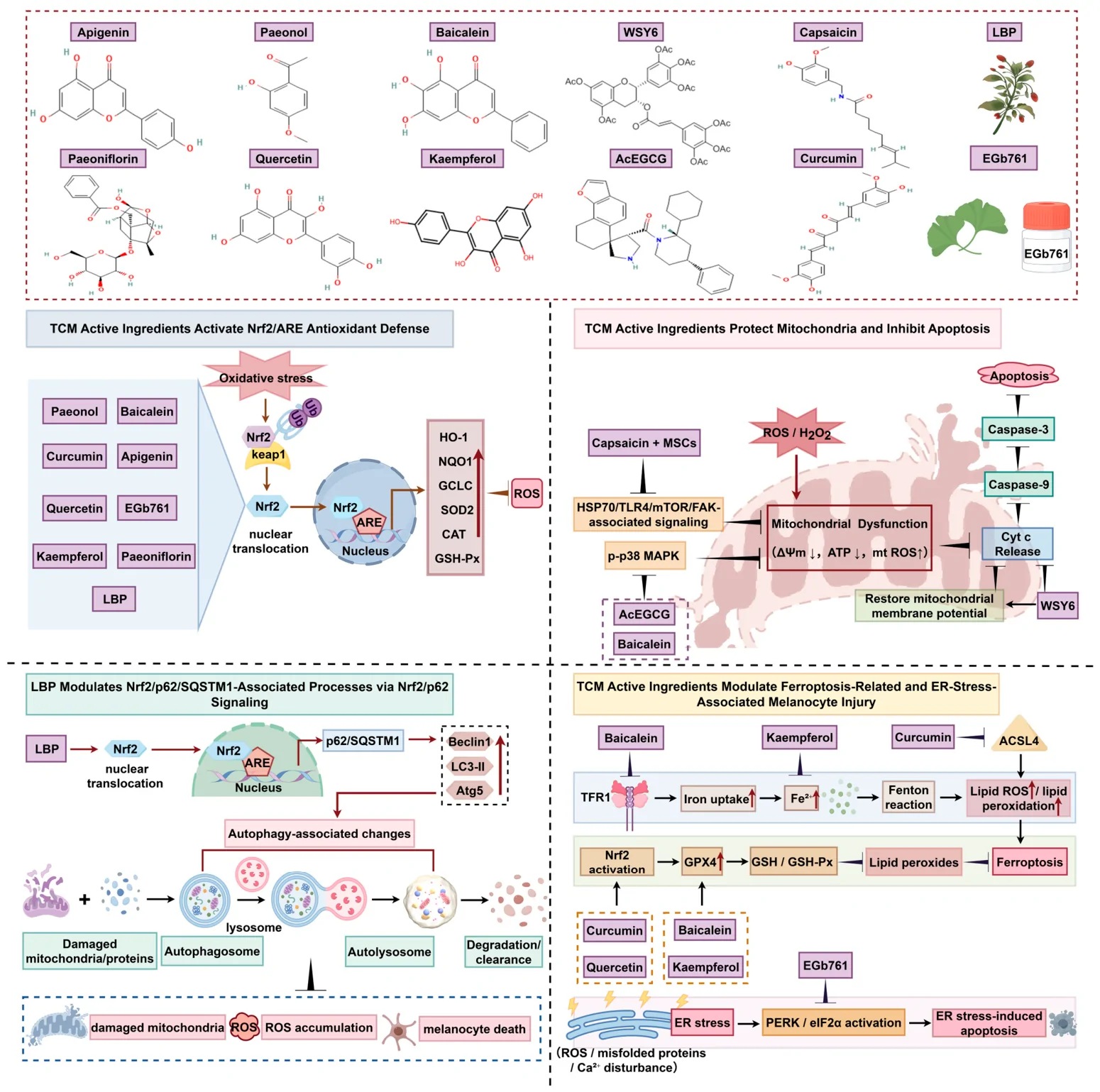
**Redox regulation and melanocyte protection by TCM-derived ingredients.** TCM-derived active ingredients protect melanocytes from oxidative stress-related injury through interconnected cytoprotective mechanisms. First, polyphenols, flavonoids, polysaccharides, and standardized botanical extracts activate the Keap1/Nrf2/ARE antioxidant defense axis, promoting Nrf2 nuclear translocation and increasing the expression of antioxidant and phase II detoxifying enzymes, including HO-1, NQO1, GCLC, SOD2, CAT, and GSH-Px. These changes reduce ROS accumulation, lipid peroxidation, and oxidative damage. Second, EGCG derivatives and baicalein preserve mitochondrial function partly by suppressing p38 MAPK phosphorylation, whereas WSY6 blocks cytochrome c release and capsaicin combined with MSCs attenuates HSP70/TLR4/mTOR/FAK-associated signaling. These effects help restore mitochondrial membrane potential, reduce mitochondrial ROS production, and inhibit caspase-9/3-dependent apoptosis. Third, LBP promotes Nrf2 nuclear translocation and activates p62/SQSTM1-associated autophagy signaling, leading to the upregulation of Beclin 1, LC3-II, and Atg5. These findings indicate modulation of autophagy-associated signaling and were accompanied by improved melanocyte survival in the reported model. Whether autophagic flux was restored requires confirmation using dynamic turnover assays. Fourth, baicalein, curcumin, quercetin, kaempferol, and EGb761-derived components modulate ferroptosis-related and ER-stress-associated melanocyte injury by modulating the TFR1/Fe^2+^/ACSL4/lipid ROS/GPX4 axis and suppressing PERK/eIF2α-related ER stress signaling. Together, these mechanisms attenuate oxidative injury, mitochondrial dysfunction, apoptosis, ferroptosis-related injury, and ER stress, thereby supporting melanocyte survival. This figure was created by Figdraw.

Beyond ferroptosis, purslane total flavones (PTFs) from *Portulaca oleracea* L. support melanocyte survival by maintaining organelle homeostasis, activating the PI3K/AKT pro-survival pathway, downregulating the B-cell lymphoma 2-associated X protein/B-cell lymphoma 2 (Bax/Bcl-2) apoptotic ratio and scavenging ROS to inhibit apoptosis [74,75,76]. Additionally, terpenes from *Ginkgo biloba* L. extract act via non-canonical pathways, suppressing H_2_O_2_-induced overactivation of ER stress signals such as the protein kinase RNA-like endoplasmic reticulum kinase/eukaryotic translation initiation factor 2 alpha (PERK/eIF2α) axis, thereby protecting melanocytes from unfolded protein response-mediated apoptosis [77]. These data suggest a multi-node protective effect; however, most evidence remains model-dependent. This distinction is important when considering whether ferroptosis- or ER stress-targeted natural compounds can be translated into clinically useful interventions for vitiligo. The redox-related mechanisms discussed in this section are summarized in Figure 1. Representative mechanistic evidence for TCM-derived active ingredients in vitiligo-related models is summarized in Table 3.

### 4.2. Promoting Melanin Synthesis and Melanosome Transport

Repigmentation requires more than the mere induction of melanin synthesis. Melanocyte stem cells (McSCs) must be activated, newly differentiated melanocytes must survive in the lesional environment, and melanosomes must be transferred efficiently to keratinocytes. Microphthalmia-associated transcription factor (MITF) and tyrosinase-family enzymes remain central to this process, but their activity depends on upstream signals such as Wingless/Int-1 (Wnt)/β-catenin, cyclic adenosine monophosphate/protein kinase A/cAMP response element-binding protein (cAMP/PKA/CREB), and MAPK pathways [78,79,80]. In this section, TCM-derived ingredients are discussed according to the stage of repigmentation they are most likely to influence.

#### 4.2.1. Activating Wnt/β-Catenin and cAMP/PKA Signaling Pathways to Promote Melanocyte Melanin Synthesis

The Wnt/β-catenin and cAMP/PKA pathways are canonical signaling cascades involved in melanogenesis. TCM-derived active ingredients may influence these processes through different signaling mechanisms.

Coumarins, such as psoralen and its derivatives from *Cullen corylifolium* (L.) Medik. [syn. *Psoralea corylifolia* L.], are primary agents in vitiligo therapy. These compounds function as both DNA-intercalating photosensitizers and potent activators of the cAMP/PKA pathway, acting in conjunction with ultraviolet (UV) radiation to upregulate TYR activity and stimulate melanocyte proliferation [81,82]. Network pharmacology combined with LC-MS/MS and experimental validation identified bergaptol as an active metabolite. Bergaptol increased p38 phosphorylation, reduced ERK phosphorylation, and enhanced MITF-mediated expression of TYR, TRP-1, and TRP-2. The melanogenic effect was validated in a PTU-induced zebrafish pigmentation model [83].

Phenolic acid derivatives from *Baccharoides anthelmintica* (L.) Moench [syn. Vernonia anthelmintica (L.) Willd.], a staple of Uyghur medicine, were associated with activation of Wnt/β-catenin-related signaling. Among these, 3,5-dicaffeoylquinic acid (3,5-diCQA) acts as a Wnt agonist by inhibiting glycogen synthase kinase 3 beta (GSK3β) activity, preventing β-catenin phosphorylation and degradation. This promotes β-catenin accumulation and nuclear translocation, where it complexes with lymphoid enhancer-binding factor/T-cell factor (LEF/TCF) to initiate MITF transcription [18]. In addition, CAM-Y7, an active extract/fraction isolated from *B. anthelmintica seeds*, contains caffeoylquinic acids, sesquiterpenoids, and flavonoids and promotes melanogenesis by activating the p38/MAPK-MAPK-activated protein kinase 2 (MAPKAPK2) axis, thereby upregulating MITF-induced tyrosinase expression. In vivo, oral administration of CAM-Y7 increased skin and hair pigmentation and attenuated chemically induced pigment loss in hydroquinone-treated mice and H_2_O_2_-treated guinea pigs, with histological analyses confirming epidermal and follicular melanogenesis [84]. This work links chemical profiling with pathway prediction, mechanistic validation, and visible pigment recovery in animal models, although it remains preclinical and requires testing in human lesions.

TCM formulas may exert multi-pathway effects through the combined actions of multiple components. Erzhi Pill, a classical formula, primarily targets the cAMP/PKA/CREB axis. It elevates intracellular cAMP levels to relieve the inhibition of protein kinase A (PKA) by its regulatory subunits, leading to CREB phosphorylation and activation, which drives MITF-dependent transcription of melanogenic enzymes and increases pigmentation-related outcomes in zebrafish [85]. Clematis tangutica (Maxim.) Korsh. leaf and stem extracts enhanced melanogenesis in B16F10 cells and zebrafish through cAMP/PKA/CREB signaling. PKA inhibition reduced the melanogenic effect. In a monobenzone-induced mouse model, the stem extract increased melanin content and TYR activity and reduced TNF-*α*, IL-6, and IL-1*β* levels [86]. Similarly, Artiri La Li Honey Pill (ALLHP) is a multi-component traditional preparation containing candidate constituents such as bakuchiol, luteolin, carvone, and psoralen. In a chemically induced depigmentation rat model, ALLHP increased epidermal melanin content, the number of melanin-containing hair follicles, and TYR levels, while reducing MDA, AChE, and MAO levels. In a zebrafish juvenile melanin-suppression model, several constituent compounds of ALLHP, including carvone, luteolin, psoralen, and bakuchiol, increased the melanin area [49]. These findings indicate antioxidant and pro-melanogenic effects in preclinical models. However, the contribution of individual constituents, the specific signaling pathways involved, pharmacological synergy, and clinical efficacy in patients with vitiligo have not been established.

#### 4.2.2. Modulating the MAPK Network to Enhance Melanin Synthesis and Transport

The mitogen-activated protein kinase (MAPK) signaling cascade serves as a central hub governing melanocyte fate. Within this network, the activation of p38 and JNK provides the primary positive drive for melanocyte differentiation and melanin synthesis. In contrast, the ERK pathway exhibits significant context-dependent effects, maintaining a dynamic balance between proliferation and pigment production. TCM active ingredients demonstrate precise targeted intervention capabilities within this complex kinase network.

**Figure 2:**
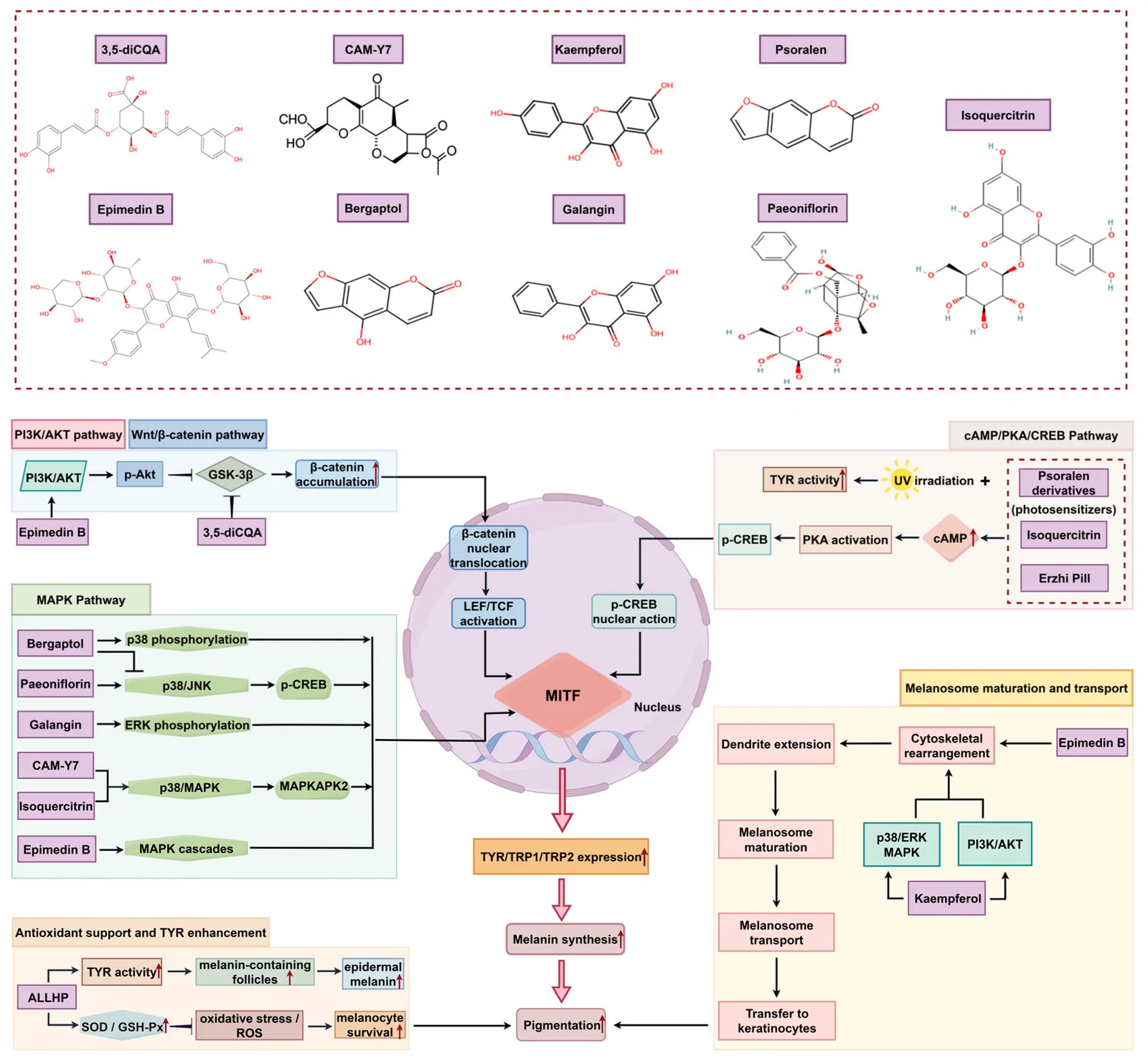
**TCM-derived ingredients regulate melanogenesis and pigmentation-related processes.** TCM-derived active ingredients may promote pigmentation-related processes by regulating melanocyte proliferation, differentiation, melanogenic enzyme expression, and melanosome maturation and transfer. Psoralen derivatives from *Cullen corylifolium* act as photosensitizers and, together with UV irradiation, activate cAMP/PKA/CREB signaling, thereby increasing MITF-dependent tyrosinase activity and melanin synthesis. Bergaptol increased p38 phosphorylation and reduced ERK phosphorylation, accompanied by increased activation of MITF-related melanogenic signaling and downstream enzyme expression in the reported model. Phenolic acid derivatives from Baccharoides anthelmintica, particularly 3,5-dicaffeoylquinic acid, activate Wnt/β-catenin signaling by inhibiting GSK3β, promoting β-catenin accumulation and LEF/TCF-mediated MITF transcription. CAM-Y7 enhances melanogenesis through the p38 MAPK/MAPKAPK2 axis, whereas Erzhi Pill activates the cAMP/PKA/CREB pathway. Galangin, isoquercitrin, paeoniflorin, epimedin B, and kaempferol further regulate MAPK, PI3K/AKT, and β-catenin-related pathways to increase MITF, TYR, TRP-1, and TRP-2 expression. Epimedin B and kaempferol also promote melanocyte dendricity, melanosome maturation, and melanosome transfer to keratinocytes. Together, these findings support pro-melanosome-transfer activities in preclinical models. This figure was created by Figdraw.

In a hydroquinone-induced C57BL/6 mouse model, apigenin attenuated depigmentation [48]. In human epidermal melanocytes, apigenin (10 μM) activated the c-KIT/Raf-1/MAPK/CREB pathway, increased MITF expression, and promoted melanogenesis, melanosome maturation, and transport. Importantly, apigenin also increased melanin content in human skin explants and promoted melanin transfer to keratinocytes [35]. Several flavonoids and flavonoid glycosides converge on MITF-centered melanogenic signaling. In PTU-treated B16F10 cells, galangin (1–2 μM for 24 h) increased melanogenesis together with p38 and JNK phosphorylation, whereas ERK1/2 was not significantly affected [87]. In B16F10 melanoma cells, isoquercitrin (10–40 μM for 24 h) increased p38 phosphorylation and further enhanced MITF activity and the expression of downstream melanogenic enzymes, including TYR and tyrosinase-related proteins 1 and 2 (TRP-1 and TRP-2), pharmacological inhibition of p38 attenuated this melanogenic response [88]. Kaempferol and epimedin B further promote melanocyte dendritic extension and melanosome transport through MAPK/PI3K/AKT and AKT/GSK3β/β-catenin signaling, respectively, thereby facilitating melanin transfer to neighboring keratinocytes [89,90]. In normal human melanocytes, Paeoniflorin (10 μg/mL for 48 h) promoted melanogenesis through the ERK/CREB-MITF pathway and increased TRP-1 expression, whereas p38 and JNK were not significantly affected [91]. The melanogenesis- and pigmentation-related mechanisms discussed in this section are summarized in Figure 2.

### 4.3. Remodeling the Immune Microenvironment

Immune modulation is relevant to vitiligo because autoreactive CD8^+^ T cells and the IFN-γ-CXCL9/10-CXCR3 axis, in which CXCL9/10 denotes C-X-C motif chemokine ligands 9/10 and CXCR3 denotes C-X-C motif chemokine receptor 3, are linked to pathways that can be targeted clinically [23,27,92,93]. JAK inhibitors show that blocking this circuit can induce repigmentation, but relapse after discontinuation suggests that pathway inhibition alone may not restore durable immune tolerance. The studies reviewed here raise a more specific question: can TCM-derived ingredients reshape this immune circuit by reducing chemokine-driven T-cell recruitment, limiting inflammatory signal transduction, or strengthening regulatory immune responses?

#### 4.3.1. Targeting T-Cell-Mediated Autoimmune Responses by Modulating Jak/Stat Signaling and Chemotactic Networks

The autoimmune destruction of melanocytes in vitiligo is largely driven by persistent activation and epidermal recruitment of CD8^+^ T cells. This process depends on pro-inflammatory cytokine signaling, particularly the interferon-γ (IFN-γ)-dependent JAK/STAT pathway, as well as downstream chemokine networks, the CXCL9/10–CXCR3 axis [26,94,95,96,97]. Demethylzeylasteral (T-96), a triterpenoid isolated from *Tripterygium wilfordii* Hook. f., has been described as a novel natural JAK inhibitor [17]. Mechanistic studies showed that T-96 may interact with the JAK3 kinase domain and inhibit JAK3/STAT5-associated signaling. In hydroquinone-induced mouse models, T-96 reduced skin-infiltrating CD8^+^ T cells and downregulated CXCL9/10 expression with efficacy comparable to the JAK inhibitor tofacitinib in this experimental model, without causing significant systemic toxicity. These findings support further evaluation of T-96 as an immunomodulatory candidate for vitiligo [17].

In addition to direct kinase inhibition, *Lycium barbarum* polysaccharides (LBPs) may modulate immunity indirectly through STAT3-dependent signaling. In vitiligo mouse models, oral LBP administration significantly inhibited STAT3 phosphorylation and reduced the expression of HSP70 and CXCL9/10, thereby suppressing upstream signals that promote CD8^+^ T-cell recruitment. LBPs also decreased the release of multiple pro-inflammatory cytokines, including interleukin-6 (IL-6), tumor necrosis factor-alpha (TNF-α), and IFN-γ, suggesting activity at several points on the STAT3-HSP70-CXCL9/10 axis [28].

Modulation of chemokine networks and innate immune activation is another way to limit autoreactive T-cell infiltration. *Rubia cordifolia* L. extract, a primary component of classical Uyghur medicine, has been shown to inhibit the IFN-γ–CXCL10–CXCR3 axis. Mechanistically, it reduces STAT1 phosphorylation and downregulates CXCL9/10 and CXCR3 expression, thereby blocking CD8^+^ T cell chemotaxis toward the epidermis. In parallel, it modulates the metabolism of pro-inflammatory lipid mediators, including lysophosphatidylcholine (LPC) and leukotriene B4 (LTB4). This broader pattern of activity lowers IFN-γ, TNF-α and interleukin-17A (IL-17A) levels and creates a less inflammatory cutaneous immune microenvironment that mitigates autoimmune damage to melanocytes [29]. Sappanone A, an isoflavonoid isolated from Caesalpinia sappan L., protected melanocytes from H_2_O_2_-induced injury and reduced HMGB1-associated inflammatory signaling in keratinocytes. In monobenzone-treated mice, it attenuated pigment loss and CD8^+^ T-cell infiltration, while transcriptomic and molecular-docking analyses implicated Wnt5a-associated noncanonical Wnt signaling as a candidate mechanism [98].

Galangin also illustrates how regulation of innate immune cells can indirectly suppress T-cell-mediated melanocyte injury. Galangin was shown to bind directly to Annexin A2 (ANXA2), as demonstrated by surface plasmon resonance (SPR), and accelerate its protein degradation in macrophages. This process suppresses macrophage overactivation and reduces the subsequent production of pro-inflammatory cytokines and chemokines. By modulating the ANXA2–macrophage–chemokine axis, galangin indirectly restricts cytotoxic T-cell recruitment into skin tissues, thereby reducing immune-mediated attack against melanocytes [99].

#### 4.3.2. Inducing Treg Differentiation to Restore Local Immune Tolerance

**Figure 3:**
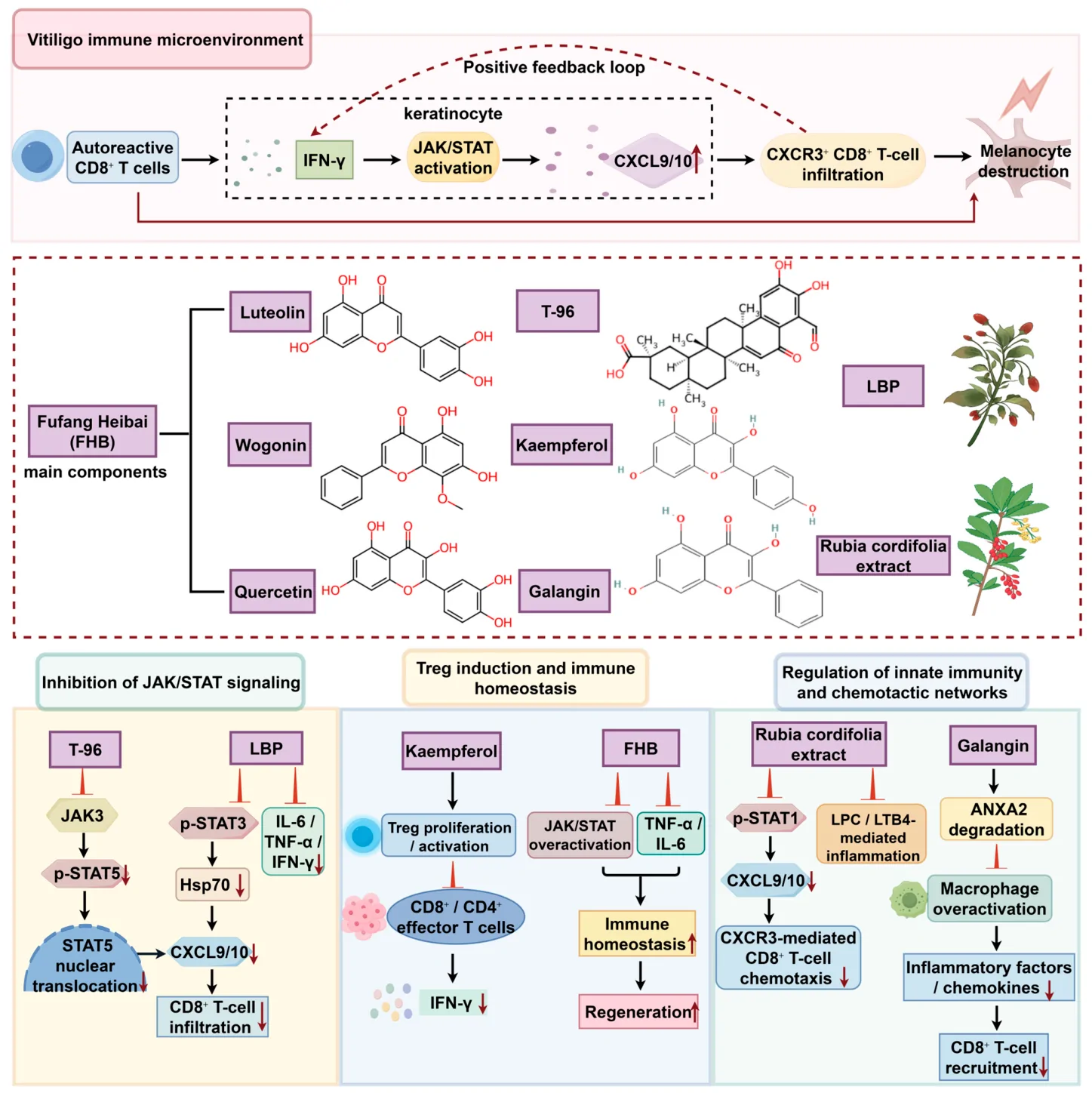
**TCM-derived immunomodulation in vitiligo.** TCM-derived active ingredients and formulas may remodel the immune microenvironment in vitiligo by modulating T-cell recruitment, inflammatory signaling, innate immune activation, and regulatory immune responses. In vitiligo lesions, IFN-γ activates JAK/STAT signaling in lesional keratinocytes, which promotes CXCL9 and CXCL10 production and recruits CXCR3^+^ cytotoxic CD8^+^ T cells, thereby contributing to melanocyte destruction. Demethylzeylasteral suppresses the JAK3/STAT5 pathway and reduces CXCL9/10-mediated CD8^+^ T-cell infiltration. Lycium barbarum polysaccharides inhibit STAT3 phosphorylation and downregulate the HSP70–CXCL9/10 axis, thereby limiting inflammatory cytokine release and T-cell recruitment. *Rubia cordifolia* L. extract attenuates the IFN-γ–STAT1–CXCL10–CXCR3 axis and modulates pro-inflammatory lipid mediators, reducing cutaneous immune activation. Galangin regulates macrophage activation through the ANXA2–macrophage–chemokine axis and indirectly limits cytotoxic T-cell infiltration. In addition, Fufang Heibai and kaempferol may restore local immune homeostasis by suppressing JAK/STAT-related inflammation and promoting regulatory T-cell responses. Together, these mechanisms suggest that TCM-derived interventions may protect melanocytes by reducing autoimmune cytotoxicity and supporting peripheral immune tolerance. This figure was created by Figdraw.

Tregs exhibit immunosuppressive function, effectively inhibiting the excessive expansion of effector CD8^+^ T cells. However, vitiligo patients show functional defects in Tregs [100,101,102]. Fufang Heibai (FHB) is a classic preparation for vitiligo. Molecular-docking analysis predicted potential interactions between STAT3 and the main active components of FuFang Heibai, including luteolin, wogonin, and quercetin. In vivo experiments demonstrate that FHB concurrently inhibits JAK/STAT overactivation, significantly downregulates the expression of TNF-α and IL-6, and promotes melanin regeneration [30]. Furthermore, kaempferol was shown to promote the proliferation and suppressive activity of regulatory T cells (Tregs) in the reported study [31]. However, whether this effect is strong enough to alter disease activity in patients remains to be determined [31]. The immunomodulatory mechanisms discussed in this section are summarized in Figure 3.

### 4.4. Potential Modulation of the Neuro–Immune–Skin Axis

A small case–control study reported psychiatric comorbidity in 90% of children and adolescents with vitiligo, compared with 20% of healthy controls [103]. Psychiatric disorders, including anxiety and depression, are also more frequently reported in adults with vitiligo [104]. Psychological stress may influence vitiligo through the brain–skin axis and associated neuroimmune mechanisms [105]. Therefore, TCM-derived ingredients that modulate these pathways may represent a potential therapeutic approach for vitiligo.

Glycyrrhizin, a triterpene saponin from *Glycyrrhiza glabra* L., is a known HMGB1 inhibitor [106]. In vitiligo, HMGB1 released from damaged melanocytes contributes to oxidative stress and inflammation, positioning glycyrrhizin as a potential mechanistic candidate [107,108]. However, direct evidence for its efficacy against vitiligo-associated depression is lacking.

Neurotrophic factors and neuroplasticity are closely associated with emotional regulation and stress adaptation [109,110,111]. Baicalein has been shown to promote hippocampal neurogenesis via the ERK/BDNF pathway, suggesting a potential role in enhancing stress-related neural resilience [112]. In chronic unpredictable mild stress models, quercetin improves cognitive and emotional behavioral parameters, modulates monoamine neurotransmitter levels, and reduces oxidative damage through the PI3K/AKT/Nrf2 signaling axis [113].

**Figure 4:**
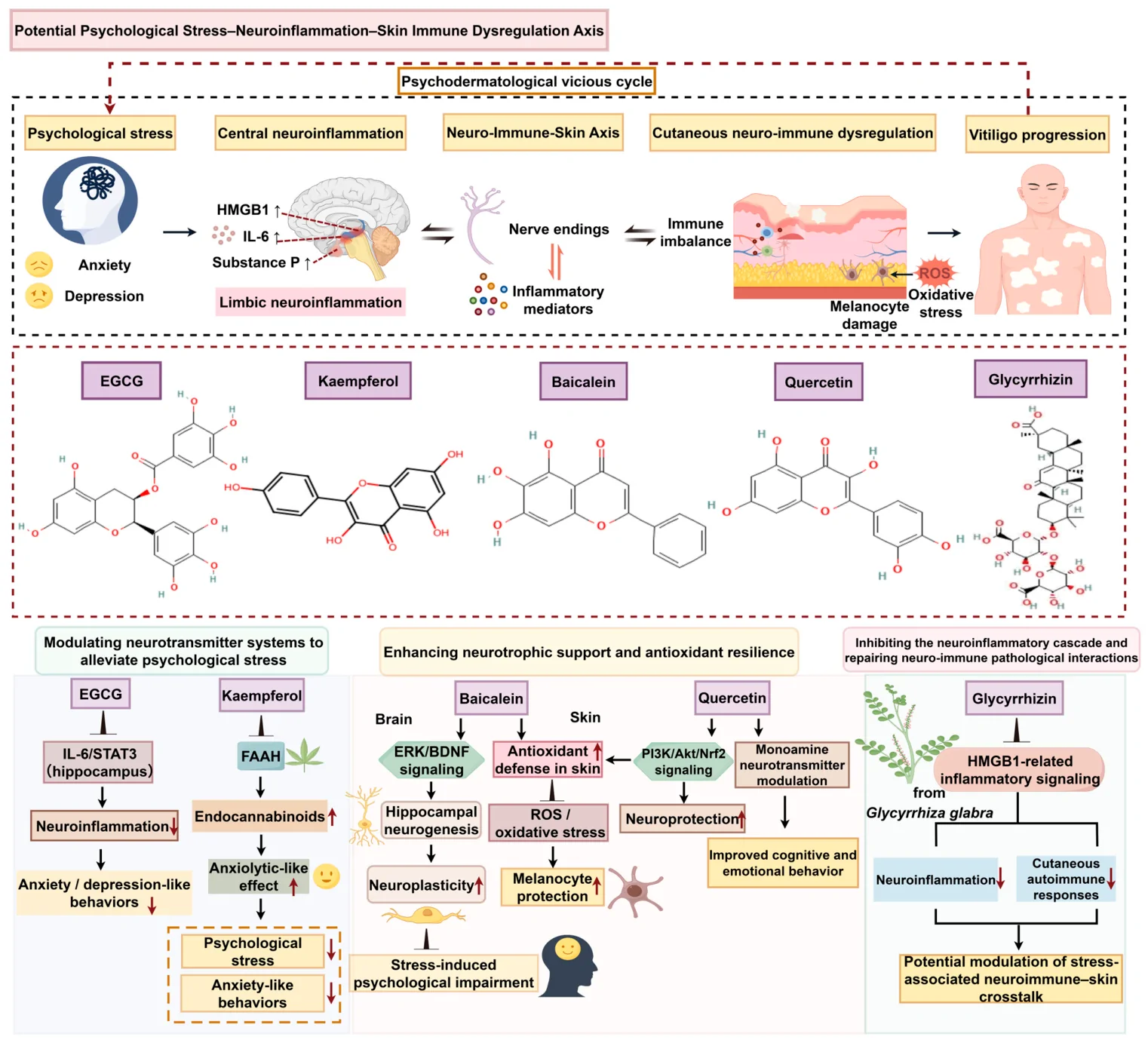
**Potential neuro–immune–skin axis regulation by TCM-derived ingredients.** Psychological stress may aggravate vitiligo through neuroinflammatory mediators such as HMGB1, IL-6, and substance P, which link central stress responses with peripheral cutaneous inflammation. Several TCM-derived compounds may act on this axis. EGCG has been reported to suppress hippocampal IL-6/STAT3 signaling, glycyrrhizin inhibits HMGB1-related inflammatory signaling, baicalein supports ERK/BDNF-mediated neurotrophic responses, quercetin activates PI3K/AKT/Nrf2-dependent antioxidant defense, and kaempferol may enhance endocannabinoid signaling through FAAH inhibition. This figure was created by Figdraw.

Neurotransmitter and inflammatory signaling may modulate stress-related symptoms. EGCG improves anxiety-like behavior by downregulating hippocampal IL-6/STAT3 signaling [114]. However, its neuropsychological effects appear to be context-dependent, and clinical evidence in vitiligo remains insufficient [115]. Kaempferol has also shown anxiolytic-like effects by inhibiting fatty acid amide hydrolase (FAAH), thereby enhancing endocannabinoid signaling [116], suggesting its potential role in stress modulation. However, most of these neurobiological findings derive from non-vitiligo stress or psychiatric models and do not demonstrate direct local skin effects or melanocyte repigmentation. Taken together, these compounds are best viewed as candidates for neuroimmune modulation rather than as proven treatments for anxiety or depression in vitiligo. The potential neuro–immune–skin axis-related mechanisms are summarized in Figure 4.

### 4.5. Translational Pharmacology, Formulation, and Exposure

Clinical translation requires adequate exposure of active compounds in the epidermis. EGCG has limited bioavailability, whereas transdermal delivery and peracetylation can increase its tissue or intracellular exposure; AcEGCG is rapidly converted to EGCG [39,117]. In vitiligo, however, topical 0.5% AcEGCG has only been reported in two patients, without measurement of lesional drug concentrations [14].

Systemic exposure may also differ from the parent compounds tested in vitro. Quercetin undergoes extensive conjugation and enterohepatic recirculation [118], glycyrrhizic acid is mainly absorbed after presystemic hydrolysis to glycyrrhetic acid [119], and native curcumin has low oral bioavailability with marked formulation-dependent differences [120]. Thus, the relevant active species in vitiligo may be the parent compound, a metabolite, or a formulation-dependent derivative.

Vitiligo-specific evidence for advanced delivery systems remains limited; psoralen/resveratrol-loaded ultradeformable liposomes have been evaluated in vitro, but human lesional exposure and clinical efficacy remain unestablished [82]. Further development should require standardized products, dose–response data, epidermal or lesional exposure, comparison of experimental with achievable concentrations, target engagement, disease-relevant efficacy, and repeat-dose safety. The routes and formulations, available pharmacokinetic evidence, relevant active species, and major evidence gaps for representative TCM-derived interventions are summarized in Table 4.

### 4.6. Safety and Toxicological Considerations

Because vitiligo often requires prolonged treatment, the safety of repeated or systemic TCM-derived interventions requires particular attention. The absence of a statistically significant difference in adverse events in small or short-duration studies should not be interpreted as evidence of established long-term safety. The major safety concerns, reported adverse effects, and evidence gaps for representative TCM-derived interventions are summarized in Table 5.

## 5. Conclusions and Future Perspectives

TCM-derived components represent a promising, mechanism-based strategy for vitiligo, a disease in which oxidative stress, impaired melanocyte regeneration, immune-mediated cytotoxicity, and neuroendocrine dysregulation contribute to disease initiation or persistence. The evidence summarized in this review indicates that diverse natural components from TCM can reinforce endogenous cytoprotective programs, counteract oxidative damage, modulate immune balance, promote melanocyte regeneration, and regulate the neuro–immune–skin axis, involving melanocytes, surrounding keratinocytes, and CD8^+^ T cells. Importantly, the translational landscape is heterogeneous. *Ginkgo biloba* L. extract, compound glycyrrhizin, turmeric preparations, Yiqi Qubai granules, and total glucosides of paeony have been supported by randomized controlled or exploratory clinical studies, typically as adjuncts to phototherapy, topical immunomodulators, or systemic therapy. In contrast, many flavonoids, phenolic acids, polysaccharides, and formula-derived constituents discussed in the mechanistic sections are still supported primarily by cell, zebrafish, or rodent data.

In the context of antioxidant defense and organelle homeostasis, paeonol, baicalein, apigenin, paeoniflorin, EGb761 and Lycium barbarum polysaccharides are most frequently linked to Nrf2/ARE signaling, mitochondrial protection, autophagy regulation and ER-stress reduction. Baicalein, curcumin, quercetin and kaempferol extend this protective profile to ferroptosis-related processes involving TFR1, GPX4, ACSL4, iron handling and lipid peroxidation. Regarding pigmentation-related processes, psoralen derivatives, 3,5-diCQA, CAM-Y7, Erzhi Pill, Artiri La Li Honey Pill, galangin, isoquercitrin, paeoniflorin, epimedin B and kaempferol have been shown to promote melanocyte proliferation, melanin synthesis, and melanosome transfer, mainly through Wnt/β-catenin, cAMP/PKA/CREB, MAPK/MITF and melanosome-transport pathways. On the immune front, demethylzeylasteral, Lycium barbarum polysaccharides, *Rubia cordifolia* L. extract, galangin, Fufang Heibai and kaempferol have been identified as potential modulators of JAK/STAT signaling, CXCL9/10-CXCR3-mediated T-cell recruitment, macrophage activation and Treg-mediated immune tolerance.

The neuro–immune–skin axis requires a more cautious interpretation. Glycyrrhizin, baicalein, quercetin, EGCG and kaempferol may influence HMGB1-related inflammation, neurotrophic signaling, monoamine neurotransmitter regulation, antioxidant defense or endocannabinoid signaling. Although these pathways are relevant to stress-related disease activity, direct evidence in vitiligo patients with psychological comorbidities remains sparse. This area should therefore be viewed as hypothesis-generating rather than clinically settled.

In this review, the effects of TCMs have been investigated across a variety of experimental models; however, the limitations of these systems must be acknowledged. B16 cells and zebrafish are useful for studying pigment synthesis but cannot model autoimmunity, while chemically induced models reflect melanocyte regeneration rather than autoimmune destruction. Immune-competent mouse models better approximate human pathogenesis but are technically challenging. Future studies should prioritize the use of human-relevant models, such as primary melanocyte–immune cell co-cultures, skin explants, and organoid systems, to better recapitulate the complex autoimmune microenvironment of vitiligo. Additionally, well-designed randomized controlled trials with standardized outcomes are urgently needed to validate the clinical efficacy and safety of TCMs and to elucidate their mechanisms of action directly in patients.

Although extensive research has elucidated the potential mechanisms of TCM-derived ingredients in vitiligo, further translational and clinical work is needed to optimize formulations, dosing, and safety for routine use. It is worth noting that certain bioactive flavonoids exhibit intense pigmentation, which may cause skin staining and affect patient adherence when used in topical formulations. Therefore, clinical translation of flavonoid-based topical preparations should consider formulation strategies to minimize this issue, such as optimizing concentrations, selecting appropriate vehicles, or developing colorless derivatives while preserving biological activity. While TCMs and their active constituents have shown promising melanogenic effects, the safety of sustained activation of Wnt/β-catenin, MITF, or PI3K/AKT pathways under chronic use warrants careful consideration. MITF is a master regulator of melanocyte survival, but its persistent overexpression has been linked to melanoma progression [127]. Similarly, sustained PI3K/AKT activation promotes cell survival and proliferation, and its dysregulation is frequently observed in various malignancies [128,129]. Although topical application of TCMs may limit systemic exposure, the long-term safety of compounds that constitutively activate these pathways in the skin has not been systematically evaluated. Future studies need to define the chemical composition of TCM formulas, verify their pharmacokinetics and lesional bioavailability, and confirm whether active compounds reach effective epidermal concentrations. Clinical trials should stratify patients by disease stage, lesion site, dominant oxidative-stress or immune phenotype and psychological burden. Outcomes should include short-term repigmentation, relapse rates, maintenance therapy requirements, safety with repeated use, and quality of life. At present, TCM-derived interventions are best positioned as potential adjunctive candidates. Their ultimate value will depend on whether mechanistic signals can be translated into reproducible, safe, and measurable clinical benefit.

## Figures and Tables

**Table 1:** **Human clinical evidence for topical and systemic TCM-derived interventions in vitiligo.**

Therapeutic Category	Compound/Formula	Source/Constituent	Evidence Type	Intervention	Main Outcomes/Implication	Ref.
Topical therapy	Curcumin/turmeric	*Curcuma longa*; curcumin-rich formulation	Randomized, double-blind placebo-controlled pilot study; systematic review of dermatological studies	Topical turmeric cream; evaluation as a potential phototherapy-compatible adjunct	Reduced lesion size and improved lesion appearance in a pilot study; antioxidant and anti-inflammatory adjunctive potential; its role as a photosensitizer for vitiligo-specific phototherapy remains unconfirmed	[11,12]
	AcEGCG	Peracetylated EGCG derivative	Clinical case reports	Topical 0.5% AcEGCG cream	Marked repigmentation; halted depigmentation progression	[14]
	EGCG	Green tea polyphenol	Clinical observation	Topical EGCG versus topical pimecrolimus	No statistically significant between-group difference in VASI improvement was detected; equivalence or non-inferiority was not established.	[13]
	*Nigella sativa* L. seed oil	Thymoquinone-rich plant oil	Clinical study	Topical *Nigella sativa* L. seed oil cream	Repigmentation was reported in facial, acral, and genital lesions; no adverse effects were reported during the 6-month study.	[40]
Systemic therapy	*Ginkgo biloba* extract	EGb; antioxidant extract	Double-blind placebo-controlled RCT; open-label pilot study	Oral EGb	Halted disease progression; induced repigmentation in some patients; systemic antioxidant support	[41,42]
	Compound glycyrrhizin	Glycyrrhizin-based immunomodulator	Meta-analysis of 39 RCTs; pediatric RCT	Oral CG plus phototherapy or immunosuppressants	Improved repigmentation; VASI improvement was observed in both groups at week 52; equivalence or non-inferiority was not established; reduced HMGB1; steroid-sparing potential	[43,44]
	Total glucosides of paeony	*Paeonia* derivatives	Retrospective clinical study	TGP plus NB-UVB and oral corticosteroid mini-pulse therapy	Improved clinical outcomes compared with NB-UVB plus oral corticosteroid mini-pulse therapy alone; enhanced repigmentation, especially in head and neck lesions; reduced serum oxidative stress markers	[45]
	Yiqi Qubai granules	TCM formula	Prospective clinical study	Oral granules plus 308 nm excimer laser	Higher response and repigmentation rates than either treatment alone; improvements in selected quality-of-life domains	[46]
	Curcumin-containing herbal beverage	Curcumin-containing multi-ingredient herbal beverage	Single case report	Long-term oral consumption	Spontaneous repigmentation occurred alongside multiple lifestyle changes; no causal effect of curcumin can be established	[47]

**Table 2:** **Preclinical evidence for topical and systemic TCM-derived interventions in vitiligo.**

Compound/Intervention	Experimental Model	Main Pharmacodynamic Findings	Translational Interpretation/Limitation	Ref.
Apigenin	Hydroquinone-induced vitiligo mouse model	Topical apigenin (1%, 2.5%, and 5%) ameliorated depigmentation, reduced oxidative stress and inflammatory markers, and increased melanin-containing hair follicles	Provides in vivo evidence of pigmentation-related and antioxidant effects; the chemically induced model does not fully reproduce autoimmune vitiligo	[48]
	PTU-induced zebrafish pigmentation model	Reversed PTU-induced pigment suppression	Supports pro-melanogenic activity in vivo, but zebrafish pigmentation does not reproduce autoimmune melanocyte destruction in human vitiligo	[35]
	Human skin explants	Increased melanin content by approximately 1.3-fold	Provides human tissue-level pharmacodynamic evidence, but does not establish clinical lesional repigmentation	[35]
Artiri La Li Honey Pill (ALLHP)	Whole ALLHP formulation: chemically induced depigmentation rat model; selected ALLHP constituents: zebrafish juvenile melanin-suppression model	Increased melanin content and tyrosinase levels and reduced malondialdehyde	Supports antioxidant and pro-melanogenic activity; findings remain preclinical and require clinical validation	[49]

**Table 3:** **Representative preclinical and mechanistic evidence for TCM-derived active ingredients in vitiligo-related models.**

Active Ingredient	Source/ Class	Experimental Model	Targets/Pathways	Mechanistic Validation	Main Effects	Mechanistic Implication	Ref.
Paeonol	Phenolic compound occurring in *Paeonia* spp.	Oxidative melanocyte injury model	Nrf2/ARE, HO-1, SOD2, CAT, GSH-Px, MDA	Nrf2 siRNA knockdown; nuclear-translocation and target-gene expression assays	Activated Nrf2 and antioxidant enzymes; reduced MDA and oxidative injury.	Antioxidant defense	[32]
Baicalein	Flavonoid	Oxidative injury and ferroptosis models	Nrf2/ARE, p38 MAPK, TFR1, GPX4	Nrf2 siRNA knockdown; p38 phosphorylation/apoptosis assays; ferroptosis-marker assays	Enhanced antioxidant defense, stabilized mitochondria, restricted iron uptake, and promoted lipid-peroxide detoxification.	Antioxidant defense; mitochondrial protection; modulation of ferroptosis-related changes	[15,64,71]
Apigenin	Flavonoid	H_2_O_2_-treated human melanocytes; hydroquinone-induced mouse model; human epidermal melanocytes	Nrf2/ARE, HO-1, SOD2, CAT, GSH-Px, c-KIT/Raf-1/MAPK/CREB	Nrf2 knockdown; histopathology/IHC; c-KIT/RSK inhibitor testing; c-KIT siRNA; MST direct-binding assay	Increased Nrf2-associated antioxidant responses; attenuated chemically induced pigment loss; increased melanogenesis and melanosome transport through p38 MAPK- and c-KIT/Raf-1/MAPK/CREB-associated signaling.	Antioxidant and pro-melanogenic activity	[33,35,48]
Kaempferol	Flavonoid	HaCaT oxidative-stress and RSL3 ferroptosis models	Nrf2/ARE, HO-1, GCLC, NQO1, GPX4, labile iron, lipid ROS	Network pharmacology/molecular docking; GPX4 siRNA knockdown	Strengthened epidermal antioxidant defense; reduced iron and lipid ROS; restored GPX4-dependent protection.	Antioxidant defense; modulation of ferroptosis-related changes	[54,73]
Paeoniflorin	Monoterpene glycoside derived from *Paeonia lactiflora* Pall.	Oxidative melanocyte injury models	JNK/Nrf2/HO-1, PDLIM1-Nrf2, RhoA/ROCK1	JNK inhibitor (SP600125); PCR-array/WB; PDLIM1 siRNA knockdown	Promoted Nrf2 nuclear translocation and HO-1; restored PDLIM1-Nrf2 signaling and reduced oxidative sensitivity.	Antioxidant defense; cytoskeletal-redox regulation	[34,55]
*Ginkgo biloba* extract EGb761	Standardized extract of *Ginkgo biloba* L. leaves, containing flavone glycosides and terpene lactones	Oxidative- or UVB-stressed skin cells	Nrf2/ARE	Nrf2 siRNA knockdown; MAPK phosphorylation/cytokine-expression assays	Increased resistance to oxidative stress and reduced UVB-induced cytotoxicity.	Antioxidant defense; tissue cytoprotection	[56,57,58]
EGCG	Green tea-derived catechin from *Camellia sinensis* (L.) Kuntze	H_2_O_2_-treated PIG1 human melanocytes	Oxidative cytotoxicity	MAPK inhibitor testing (injury model); p38 phosphorylation/ROS/viability assays	Dose-dependently attenuated H_2_O_2_-induced oxidative injury and improved melanocyte survival	Antioxidant defense; cytoprotection	[62]
AcEGCG	Peracetylated semisynthetic derivative of EGCG from *Camellia sinensis* (L.) Kuntze	H_2_O_2_-treated human epidermal melanocytes	ROS, p38 MAPK, mitochondrial membrane potential	p38 phosphorylation; ROS/mitochondrial/apoptosis assays	Reduced ROS generation and p38 MAPK activation, restored mitochondrial membrane potential, and inhibited apoptosis.	Mitochondrial protection; apoptosis inhibition	[63]
WSY6	Semisynthetic caffeic acid derivative	H_2_O_2_-treated PIG1 human melanocytes	ROS, p38/ERK/JNK MAPK, cytochrome c, caspase-9/3	MAPK inhibitor testing (injury model); MAPK phosphorylation; cytochrome-c/caspase assays	Reduced oxidative stress, MAPK activation, cytochrome c release, and mitochondria-dependent apoptosis.	Antioxidant defense; mitochondrial protection	[62]
Capsaicin combined with MSCs	Capsaicinoid derived from *Capsicum* spp.; evaluated in combination with mesenchymal stem cells	PIG3V mitochondrial dysfunction model	HSP70/TLR4/mTOR/FAK	HSP70 overexpression; co-immunoprecipitation; RT-qPCR/WB	Improved mitochondrial dysfunction-related outcomes and modulated HSP70/TLR4/mTOR/FAK-associated signaling under combined capsaicin and MSC treatment.	Mitochondrial homeostasis; organelle repair	[68]
*Lycium barbarum* polysaccharide	Polysaccharide fraction from *Lycium barbarum* L.	H_2_O_2_-induced melanocyte injury model	Nrf2/p62, Beclin 1, LC3-II, Atg5	Rapamycin/3-MA modulation; Nrf2 overexpression/knockdown; RT-PCR/WB/TEM	Promoted Nrf2 translocation and autophagy; enhanced proliferation and survival.	Antioxidant defense; autophagy regulation	[69]
Curcumin	Polyphenolic diarylheptanoid derived from *Curcuma longa* L.	Erastin-induced melanocyte ferroptosis model	Nrf2/HO-1, GPX4, ACSL4, GSH, SOD	Network pharmacology/molecular docking; Fer-1 rescue; Nrf2 inhibitor (ML385)	Activated antioxidant signaling; modulated ferroptosis-related proteins; reduced iron accumulation and lipid peroxidation.	Antioxidant defense; ferroptosis inhibition	[72]
Quercetin	Flavonoid; Yubai Wan constituent	Hydroquinone-induced guinea pig and B16 models	Nrf2/GPX4	Metabolomic/transcriptomic analysis; Nrf2 inhibitor (ML385); ferroptosis-marker assays	Activated Nrf2/GPX4 and reduced lipid peroxidation; Nrf2 inhibition with ML385 partially attenuated the protective effect.	Antioxidant defense; ferroptosis inhibition	[16]
Purslane total flavones (PTF)	Total flavonoid extract from *Portulaca oleracea* L.	Oxidative stress and apoptosis models	PI3K/AKT, Bax/Bcl-2, ROS	Network pharmacology/molecular docking; PI3K inhibitor (LY294002); WB/functional assays	Scavenged ROS, preserved organelles, activated survival signaling, and reduced apoptosis.	Antioxidant defense; apoptosis inhibition	[74]
Bilobalide	Sesquiterpene trilactone from *Ginkgo biloba* L.	H_2_O_2_-induced ER-stress model	PERK/eIF2alpha	CAT/GPx1 mRNA; eIF2α phosphorylation/CHOP expression; ROS/apoptosis assays	Suppressed excessive ER-stress signaling and unfolded-protein-response-mediated apoptosis.	ER-stress inhibition; apoptosis inhibition	[77]
Artiri La Li Honey Pill (ALLHP)	Traditional honey-pill formulation containing multiple herbal constituents; active compounds include bakuchiol, luteolin, carvone, and psoralen	Chemically induced depigmentation rat model; zebrafish juvenile melanin-suppression model for selected ALLHP constituents	TYR, MDA, AChE, MAO; specific signaling pathway not established	Biochemical/histological phenotyping; zebrafish constituent screening	Increased epidermal melanin content, melanin-containing hair follicles, and TYR levels; reduced MDA, AChE, and MAO; selected constituent compounds increased melanin area in zebrafish.	Antioxidant and pro-melanogenic activity	[49]

**Table 4:** **Translational pharmacology of representative TCM-derived interventions.**

Candidate	Route/Formulation	Available Pharmacokinetic and Formulation Evidence	Relevant Active Species	Main Evidence Gap	Ref.
EGCG/ AcEGCG	Topical; 0.5% AcEGCG cream	Transdermal EGCG (50 mg/kg) reached 1365.7–121.0 ng/g in epidermis and 411.2–42.6 ng/g in dermis (plasma Cmax: 44.5 ng/mL); AcEGCG increased plasma EGCG AUC ~2.4-fold (465.0 vs. 194.6 μg·min/mL).	AcEGCG → EGCG	Human lesional concentration unknown	[14,39,117]
Curcumin/ turmeric	Topical and oral formulations	Low oral bioavailability; formulation-dependent exposure; no vitiligo-specific plasma or skin PK data.	Parent compound and metabolites	Vitiligo-specific skin exposure unknown	[11,120]
Quercetin	Mainly preclinical for vitiligo; human oral PK available	Human oral quercetin showed high apparent clearance (CL/F ≈ 3.5 × 10^4^ L/h) and a terminal t1/2 of ~3.5 h, with extensive conjugation and enterohepatic recirculation.	Parent and conjugated metabolites	Experimental concentrations versus achievable skin exposure unknown	[118]
Compound glycyrrhizin	Oral; including 50–150 mg/d in vitiligo	Oral glycyrrhizic acid undergoes extensive presystemic conversion to glycyrrhetic acid; quantitative exposure in vitiligo skin has not been established.	Glycyrrhetic acid is a major relevant metabolite	Parent/metabolite exposure in vitiligo skin unknown	[43,44,119]
Psoralen/ resveratrol	Ultradeformable liposomes	Ultradeformable liposomes showed ~74% psoralen and ~77% resveratrol entrapment efficiency in vitro; human lesional exposure has not been established.	Formulation-delivered parent compounds	No human lesional PK or clinical efficacy	[82]

**Table 5:** **Safety considerations for representative TCM-derived interventions relevant to vitiligo.**

Intervention	Route/Exposure	Main Safety Concerns	Contraindications/Interactions	Monitoring	Exposure/Evidence Gap	Ref.
Compound glycyrrhizin	Oral; repeated systemic use	Hypertension, edema, hypokalemia, pseudoaldosteronism	Caution in cardiovascular or renal disease and with potassium-lowering drugs	Blood pressure, edema, serum K^+^, renal function	Long-term safety in vitiligo remains insufficiently characterized	[43,44,121]
Psoralen-containing preparations	Topical or oral; particularly with UVA	Phototoxicity, erythema and burns; cumulative PUVA exposure is a long-term concern	Photosensitivity disorders; concomitant photosensitizing drugs	Phototoxic reactions, cumulative UVA/PUVA exposure, skin examination	Long-term risk from repeated herbal psoralen exposure in vitiligo is incompletely defined	[81,82,122,123]
Ginkgo biloba extract	Oral	Potential bleeding and drug-interaction concerns	Caution with anticoagulant or antiplatelet therapy	Medication review and clinical signs of bleeding	Controlled studies of standardized extracts have not consistently shown increased bleeding, but long-term vitiligo data are limited	[41,42,124]
Demethylzeylasteral (T-96)/Tripterygium-derived compounds	T-96: preclinical systemic exposure	Tripterygium preparations are associated with gastrointestinal, hepatic, renal, hematologic and reproductive toxicity	Pregnancy or reproductive planning; hepatic or renal disease; caution with immunosuppressive therapy	CBC, liver and renal function; reproductive toxicity assessment	Toxicity of Tripterygium preparations should not be directly attributed to isolated T-96; chronic and reproductive safety of T-96 remains undefined	[17,125]
Complex multi-component formulas	Oral or topical, formulation dependent	Constituent-dependent toxicity, interactions and product variability	Polypharmacy; anticoagulant, antiplatelet or immunosuppressive therapy	Product- and constituent-specific monitoring	Standardization, interaction studies and long-term exposure data are often insufficient	[126]

## Data Availability

No new data were created or analyzed in this study. Data sharing is not applicable to this article.

## Abbreviations

3,5-diCQA	3,5-dicaffeoylquinic acid
AcEGCG	peracetylated epigallocatechin-3-gallate
ACSL4	acyl-CoA synthetase long-chain family member 4
ALLHP	Artiri La Li Honey Pill
ANXA2	annexin A2
BDNF	brain-derived neurotrophic factor
cAMP	cyclic adenosine monophosphate
CAT	catalase
CG	compound glycyrrhizin
CREB	cAMP response element-binding protein
CXCL9	C-X-C motif chemokine ligand 9
CXCL10	C-X-C motif chemokine ligand 10
CXCR3	C-X-C motif chemokine receptor 3
EGb	Ginkgo biloba extract
EGb761	standardized *Ginkgo biloba* L. extract
EGCG	epigallocatechin-3-gallate
ER	endoplasmic reticulum
ERK	extracellular signal-regulated kinase
FAAH	fatty acid amide hydrolase
FHB	Fufang Heibai
GPX4	glutathione peroxidase 4
GSH-Px	glutathione peroxidase
GSK3β	glycogen synthase kinase 3 beta
HMGB1	high-mobility group box 1
HO-1	heme oxygenase-1
HSP70	heat shock protein 70
IFN-γ	interferon-gamma
IL-6	interleukin-6
IL-17A	interleukin-17A
JAK	Janus kinase
JAK3	Janus kinase 3
JNK	c-Jun N-terminal kinase
LBP	Lycium barbarum polysaccharide
MAPK	mitogen-activated protein kinase
MAPKAPK2	MAPK-activated protein kinase 2
McSCs	melanocyte stem cells
MDA	malondialdehyde
MITF	microphthalmia-associated transcription factor
mTOR	mechanistic target of rapamycin
NB-UVB	narrowband ultraviolet B
Nrf2	nuclear factor erythroid 2-related factor 2
PERK	protein kinase RNA-like endoplasmic reticulum kinase
PI3K/AKT	phosphoinositide 3-kinase/protein kinase B
PKA	protein kinase A
PTU	phenylthiourea
ROS	reactive oxygen species
SOD	superoxide dismutase
STAT1	signal transducer and activator of transcription 1
STAT3	signal transducer and activator of transcription 3
STAT5	signal transducer and activator of transcription 5
TCM	traditional Chinese medicine
PTF	purslane total flavones
TFR1	transferrin receptor 1
TGP	total glucosides of paeony
TLR4	Toll-like receptor 4
TNF-α	tumor necrosis factor-alpha
Treg	regulatory T cell
TRP	tyrosinase-related protein
TYR	tyrosinase
UVB	ultraviolet B
VASI	Vitiligo Area Scoring Index
Wnt/*β*-catenin	Wingless/Int-1/*β*-catenin signaling pathway
